# A Multivalent ICAM-1 Binding Nanoparticle which Inhibits ICAM-1 and LFA-1 Interaction Represents a New Tool for the Investigation of Autoimmune-Mediated Dry Eye

**DOI:** 10.3390/ijms21082758

**Published:** 2020-04-15

**Authors:** Pang-Yu Hsueh, Yaping Ju, Adrianna Vega, Maria C. Edman, J. Andrew MacKay, Sarah F. Hamm-Alvarez

**Affiliations:** 1 Department of Pharmacology and Pharmaceutical Sciences, School of Pharmacy, University of Southern California, Los Angeles, CA 90033, USA; pangyuhs@gmail.com (P.-Y.H.); yju@usc.edu (Y.J.); vega@usc.edu (A.V.); 2 Department of Ophthalmology, USC Roski Eye Institute, University of Southern California, Los Angeles, CA 90033, USA; edman@usc.edu; 3 Department of Biomedical Engineering, University of Southern California, Los Angeles, CA 90089, USA

**Keywords:** elastin-like polypeptide, intercellular adhesion molecule-1, Sjögren’s syndrome, dry eye, lymphocyte function-associated 1 antigen

## Abstract

The autoimmune disorder, Sjögren’s syndrome (SS), is characterized by lymphocytic infiltration and loss of function of exocrine glands such as the lacrimal gland (LG) and salivary gland. SS-associated changes in the LG are associated with the development of autoimmune-mediated dry eye disease. We have previously reported the accumulation of intercellular adhesion molecule 1 (ICAM-1) in the LG of Non-Obese Diabetic (NOD) mice, a murine model of autoimmune-mediated dry eye in SS, in both LG acinar cells and infiltrating lymphocytes. ICAM-1 initiates T-cell activation and can trigger T-cell migration through binding to lymphocyte function-associated 1 antigen (LFA). To modulate this interaction, this study introduces a new tool, a multivalent biopolymeric nanoparticle assembled from a diblock elastin-like polypeptide (ELP) using the S48I48 (SI) ELP scaffold fused with a mouse ICAM-1 targeting peptide to form IBP-SI. IBP-SI forms a multivalent, monodisperse nanoparticle with a radius of 21.9 nm. Unlike the parent SI, IBP-SI binds mouse ICAM-1 and is internalized by endocytosis into transfected HeLa cells before it accumulates in lysosomes. In vitro assays measuring lymphocyte adhesion to Tumor Necrosis Factor TNF-α-treated bEnd.3 cells, which express high levels of ICAM-1, show that adhesion is inhibited by IBP-SI but not by SI, with IC_50_ values of 62.7 μM and 81.2 μM, respectively, in two different assay formats. IBP-SI, but not SI, also blocked T-cell proliferation in a mixed lymphocyte reaction by 74% relative to proliferation in an untreated mixed cell reaction. These data suggest that a biopolymeric nanoparticle with affinity for ICAM-1 can disrupt ICAM-1 and LFA interactions in vitro and may have further utility as an in vivo tool or potential therapeutic.

## 1. Introduction

Sjögrens’ syndrome (SS) is an autoimmune disease associated with lymphocytic inflammation of exocrine secretory tissues including the lacrimal gland (LG) and salivary gland (SG), reduction of glandular secretory capacity and consequent development of severe dry eye and dry mouth [1]. The autoimmune inflammation in SS may also affect other organ systems such as the kidney, liver and brain, while SS patients have a significantly increased incidence of B-cell lymphoma [2]. Currently, there is no effective and specific therapy for the autoimmune-mediated dry eye associated with SS due to an incomplete understanding of disease pathology in the LG [3,4], and also due to the inability to target therapies which may reduce lymphocytic infiltration to the LG. As is typical of other autoimmune disease patients, patients with SS are often treated with topical, oral, or intravenous immunomodulatory agents, including steroids and cyclosporine A, to suppress systemic and local T-cell proliferation [2,5,6]. Work in a murine model of SS has shown that the immunomodulatory agent, rapamycin, also has potential in treating the autoimmune inflammation of LG and in improving tear flow [7,8]. Herein, we report on a new tool, the use of a multivalent nanoparticle that is capable of inhibiting interactions critical for immune cell migration into the LG. The target of this multivalent nanoparticle is intercellular adhesion molecule-1 (ICAM-1, or CD54).

ICAM-1 is a cell-surface glycoprotein member of the immunoglobulin (Ig) superfamily, and consists of five extracellular Ig-like domains, a transmembrane domain, and a short cytoplasmic domain [9]. It exists in two isoforms: membrane-bound and soluble ICAM-1 (sICAM-1) [10]. ICAM-1 is constitutively expressed at low levels on the surface of most cells; its upregulation is induced by interleukin-1 (IL-1), interferon-*γ*, and tumor necrosis factor (TNF)-*α* [11] in response to inflammatory stimuli. As the homing receptor for leukocytes and macrophages, ICAM-1 is involved in lymphocyte migration, co-activation of T- and B -cells, and leukocyte extravasation into lymphoid and inflamed non-lymphoid tissues through interactions with β_2_ integrin lymphocyte function-associated antigen-1 (LFA-1, α_L_β_2_, or CD11a/CD18) and macrophage 1 antigen [12]. ICAM-1 expression is significantly correlated with the progression of many inflammatory diseases. For example, monitoring the concentration of circulating sICAM-1 can improve the prediction of diseases such as atherosclerosis [13,14], diabetes [15,16], and cerebral malaria [17].

In terms of SS, biopsies from the conjunctiva, LG, and SG of human and SS-susceptible animal models (e.g., mouse, rat, and canine) exhibit lymphocytic infiltration with increased expression of various inflammatory and immune activation markers such as ICAM-1, LFA-1, and major histocompatibility complex class II antigens [18,19]. In a murine model of the autoimmune-mediated dry eye characteristic of SS, the male Non-Obese Diabetic NOD mouse, ICAM-1 is highly expressed in the LG, both in LG acinar cells (LGAC) and in infiltrating immune cells [20]. This finding suggests that ICAM-1 might constitute a target for the disruption of immune cell homing to the LG. Studies targeting ICAM-1/LFA-1 interactions as a strategy to develop novel anti-inflammatory therapies have mainly focused on other immunoregulatory conditions, such as graft rejection, atopic dermatitis, psoriasis, and rheumatoid arthritis [21,22,23]. However, an ophthalmic solution, 5% Lifitegrast (Xiidra^®^), is also approved for the treatment of dry eye. This novel integrin antagonist mimics the binding epitope of ICAM-1, thus reducing the binding of LFA-1 to endogenous ICAM-1 and inhibiting downstream inflammation [24].

Our group recently showed that the addition of a single ICAM-1 binding peptide (IBP) to a protein nanocarrier administered intravenously can transiently increase the accumulation of this nanocarrier in the LG in the NOD mouse model of autoimmune-mediated dry eye, relative to the untargeted nanocarrier [20]. We hypothesized that a nanoparticle containing multiple copies of IBP might be able to functionally disrupt ICAM-1 and LFA interactions in the LG. As the first step in testing this hypothesis, an anti-mouse IBP [25] was fused to an elastin-like polypeptide (ELP) biopolymer to assemble a nanoparticle. Mimicking the repetitive hydrophobic domains of human tropoelastin, ELPs are composed of a repeating pentameric motif (Val-Pro-Gly-Xaa-Gly)_n_, where Xaa can be substituted with amino acids that possess different hydrophobicity or hydrophilicity, thus changing the assembly properties [26]. ELPs phase separate above a lower critical solution temperature, which can be tuned by the selection of Xaa and n [26,27]. The backbone ELP used in this study was a diblock copolymer with 48 serine (S48) and 48 isoleucine (I48) guest residues (S48I48, SI). SI has previously been shown to assemble a nanoparticle capable of sequestering hydrophobic drugs such as rapamycin for therapeutic administration in vivo in a mouse model of SS [7,28]. Expressed and purified from *E. coli*, the diblock copolymer IBP-SI construct assembles into a stable nanoparticle with biophysical properties comparable to the parent SI, but with the additional ability to bind and to be internalized by the mouse ICAM-1 receptor expressed in HeLa cells, as well as to engage with the endogenous human receptor. Finally, IBP-SI, but not SI, impairs lymphocyte adhesion to cultured bEnd.3 endothelial cells expressing high levels of ICAM-1, as well as T-cell proliferation in response to antigen presentation in a mixed cell reaction.

## 2. Results

### 2.1. IBP-SI Forms Nanoparticles at Physiological Temperature

**Figure 1:**
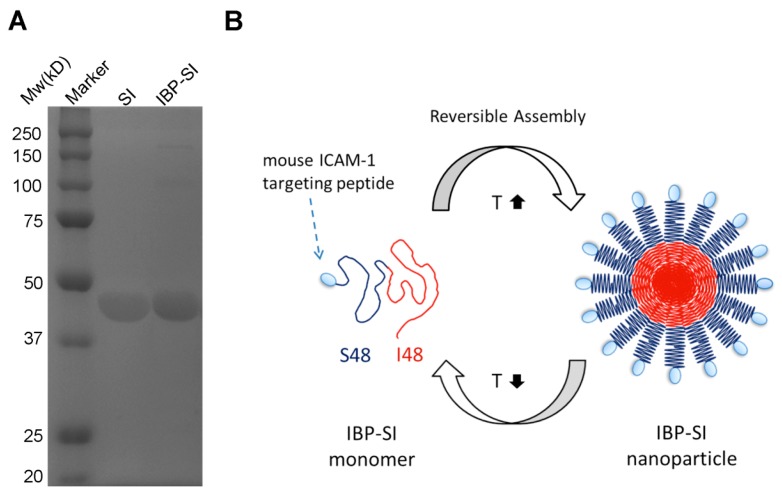
Characterization and design of an elastin-like polypeptide (ELP) nanoparticle to target intercellular adhesion molecule 1 (ICAM-1). (**A**) Purified ELPs were resolved on 4–12% SDS-PAGE gels and stained with 10% (*w*/*v*) copper chloride, revealing bands consistent with the estimated molecular weight (Mw) of SI (39.6 kD) and IBP-SI (41.5 kD). (**B**) An ICAM-1 binding peptide (IBP) was appended to the amino-terminus of a diblock copolymer consisting of the hydrophilic ELP (VPGSG)_48_, illustrated as S48 and shown in dark blue, and the hydrophobic ELP (VPGIG)_48_, illustrated as I48 and shown in red, designated IBP-SI. IBP-SI assembles into nanoparticles at physiological temperatures (37 °C).

The genetically engineered protein–polymer termed IBP-SI was expressed in BLR(DE3) competent *E. coli* and purified from *E. coli* lysates by the induction of ELP-mediated phase separation. IBP-SI consists of a mouse ICAM-1 targeting peptide, which binds murine ICAM-1 and inhibits ICAM-1-mediated intercellular adhesion [25]. IBP was linked to the N-terminus of an ELP called SI, which is comprised of an N-terminal hydrophilic peptide motif, (Val-Pro-Gly-Ser-Gly)_48_, and a C-terminal hydrophobic peptide motif, (Val-Pro-Gly-Ile-Gly)_48_ (Table 1). Like SI, IBP-SI was anticipated to form a core-shell nanoparticle above its critical micelle (first) temperature, *T_t_*_1_, (Figure 1B) and a bulk coacervate above its higher (second) transition temperature, *T_t_*_2_. Gel electrophoresis using SDS-PAGE and staining with 10% (*w*/*v*) copper chloride confirmed the molecular mass and purity of the IBP-SI and SI ELPs, showing that the purity of IBP-SI was 87.92% compared to 92.64% of its SI counterpart (Figure 1A). Molecular weights of SI and IBP-SI were confirmed by matrix-assisted laser desorption/ionization time of flight mass spectrometry and were 39.5 kDa and 41.4 kDa, respectively, within 0.3% of their expected weights (Table 1; Figure 1A).

**Figure 2:**
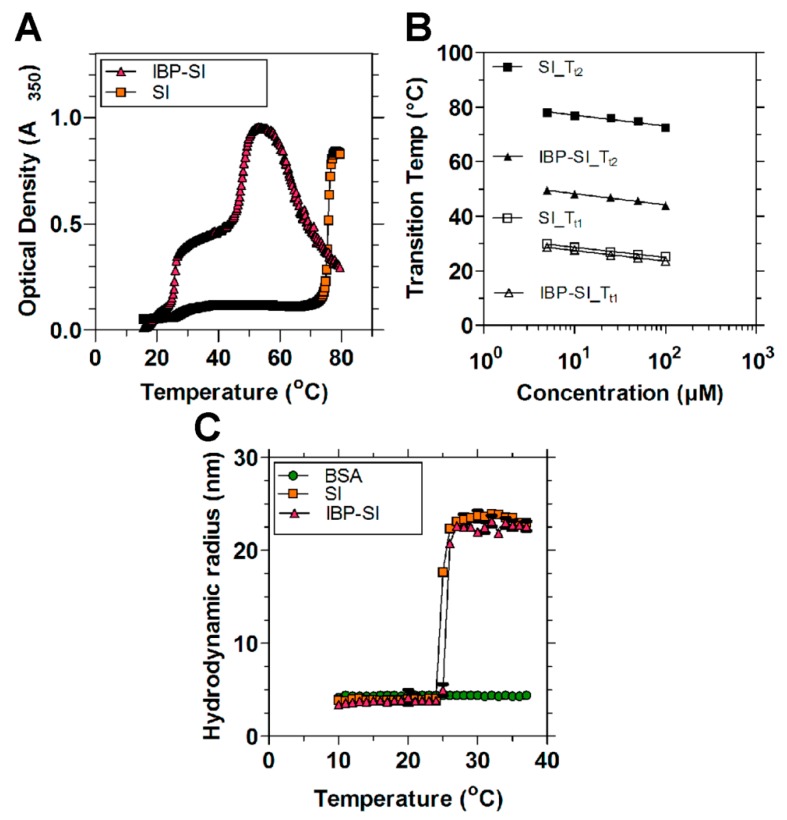
ELP protein–polymers with or without ICAM-1-targeting peptides form nanoparticles at physiological temperature. (**A**) Temperature-induced assembly of ELPs was determined by measurement of optical density (OD_350_) as a function of temperature and concentration. Both SI and IBP-SI (25 μM in PBS) have two obvious inflections. *T_t_*_1_ is consistent with the assembly of the hydrophobic I48, whereas *T_t_*_2_ is associated with the bulk phase separation of hydrophilic S48. Diblock ELPs assemble into nanoparticles between *T_t_*_1_ and *T_t_*_2_. At this concentration, SI assembles into nanoparticles between 25.5 °C and 73.7 °C and IBP-SI assembles nanoparticles between 25.7 °C and 46.9 °C. (**B**) The concentration–temperature phase diagrams for SI and IBP-SI follow a log-linear relationship. The *T_t_s* of each ELP were fitted to the equation *T_t_* = *m* Log_10_ (*C_ELP_*) + *b*, where *m* is the slope, *C_ELP_* is the concentration, and *b* is the transition temperature at 1 μM (Appendix A Table A1). (**C**) DLS was used to measure the self-assembly and hydrodynamic radius of ELP nanoparticles (25 μM in PBS) up to 37 °C. The ICAM-1 binding peptide has minimal influence on the assembly and radius of ELP nanoparticles. Bovine serum albumin (BSA) was used as an internal control. At 37 °C, SI and IBP-SI form nanoparticles with a hydrodynamic radius of 23.6 ± 0.4 nm and 21.9 ± 0.6 nm, respectively. Data are presented as mean ± SD.

The thermal transition behavior (*T_t_*_1_ and *T_t_*_2_) of these ELPs was determined using UV-vis spectrophotometry by monitoring the optical density at 350 nm as a function of temperature in PBS. SI exhibited two steep thermal responses at 25.5 °C (*T_t_*_1_) and 73.8 °C (*T_t_*_2_), conferred by the hydrophobic (Val-Pro-Gly-Ile-Gly)_48_ and the hydrophilic (Val-Pro-Gly-Ser-Gly)_48_ motifs, respectively (Table 1; Figure 2A). In general, guest residues (Xaa) that are more hydrophobic confer a lower transition temperature relative to those that are hydrophilic. The first phase separation at *T_t_*_1_ involves a transition from soluble monomers to supramolecular micelles. Upon heating, nanoparticle assembly causes an abrupt increase in optical density. SI remains a stable nanoparticle between *T_t_*_1_ and *T_t_*_2_, undergoing a bulk phase transition to coacervate when the temperature reaches *T_t2_*. Similar to SI, IBP-SI showed two obvious phase transitions at 25.7 °C (*T_t_*_1_) and 46.9 °C (*T_t_*_2_). Both SI and IBP-SI exhibit similar *T_t_*_1_; however, they have distinct *T_t_*_2_. The reduction in *T_t_*_2_ for IBP-SI compared to SI may result from the adhesion between IBP-decorated nanoparticles. The concentration dependence of *T_t_* for SI and IBP-SI is log-linear (Figure 2B), in accordance with many other reported ELP fusions [27,29,30]. The hydrodynamic radii of SI and IBP-SI were also determined using dynamic light scattering (DLS) at 37 °C. SI and IBP-SI exist as nanoparticles between *T_t_*_1_ and *T_t_*_2_, with radii of 23.6 ± 0.4 nm and 21.9 ± 0.6 nm, respectively (Table 1; Figure 2C). These results suggest that the addition of mouse ICAM-1 binding peptide to SI minimally influences the critical micelle temperature (*T_t_*_1_) and particle size of SI at physiological temperatures.

### 2.2. IBP-SI Nanoparticles Target ICAM-1 In Vitro

**Figure 3:**
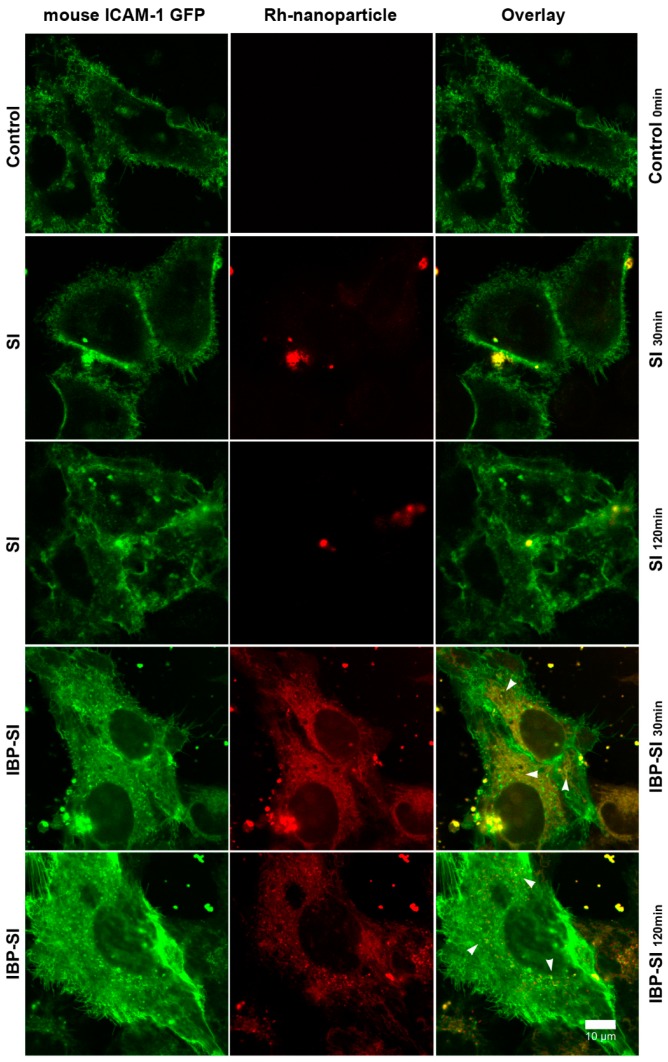
IBP-SI is internalized in HeLa cells expressing mouse ICAM-1. HeLa cells transfected with mICAM-1 GFP were incubated with rhodamine (Rh)-labeled ELPs at 37 °C for 30 min or 120 min prior to imaging using confocal fluorescence microscopy. At comparable time points, IBP-SI-treated cells displayed more accumulation of red puncta within vesicular internal compartments than those incubated with SI. ICAM-1 expressing HeLa cells without ELP treatment was used as a negative control. This experiment was repeated at least twice. Green, mouse ICAM-1 GFP; red, ELP nanoparticles; white arrowheads, internalized ELP nanoparticles. Scale bar = 10 µm.

ICAM-1 transports its ligands to lysosomes via ICAM-1 mediated endocytosis [31]. In endothelial cells, ICAM-1 binding to cargo triggers nonclassical endocytosis and delivery to lysosomes [32]. To determine if IBP-SI can selectively target ICAM-1 and be internalized through ICAM-1 dependent endocytosis into cells, we used HeLa cells transfected with and expressing fluorescently tagged mouse ICAM-1 as an in vitro model. Confocal fluorescence microscopy was utilized to track the internalization of rhodamine-labeled (rh) materials. The cells were incubated with 30 µM of rh-ELPs at 37 °C for 30 min and 120 min before analysis. Cells incubated with rh-SI showed little to no fluorescent signal in the cytoplasm, while cells incubated with rh-IBP-SI showed significant punctate intracellular accumulation, suggesting that IBP-SI was internalized via ICAM-1 mediated endocytosis (Figure 3).

**Figure A2:**
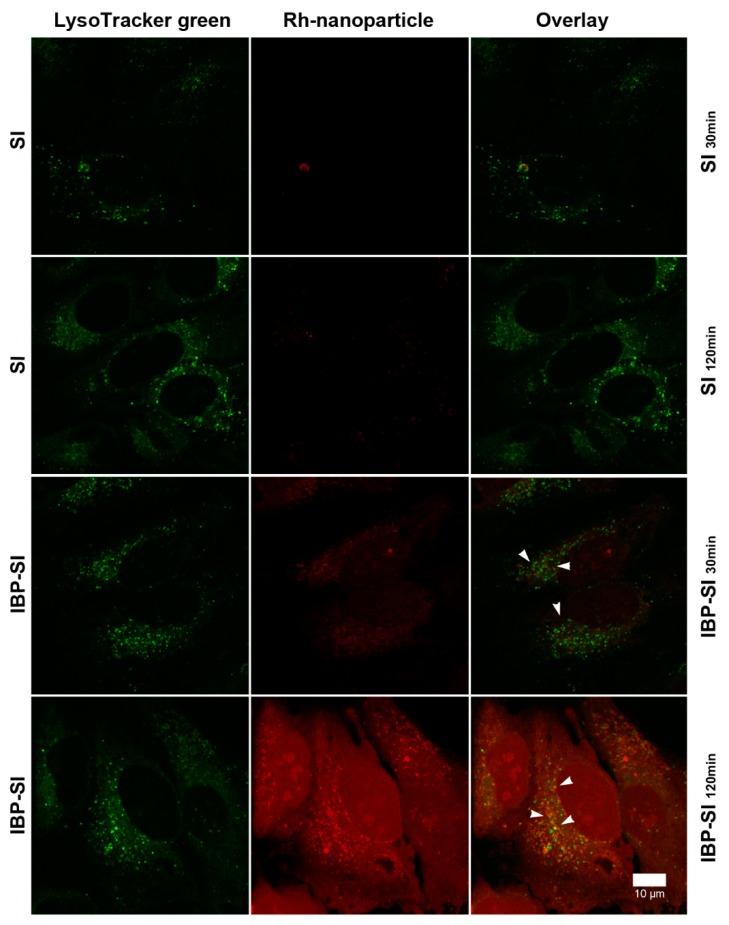
IBP-SI exhibits a cross-species targeting activity to human ICAM-1. HeLa cells were treated with rhodamine-labelled ELPs at 37 °C for 30 min or 120 min prior to imaging using confocal fluorescence microscopy. LysoTracker Green (70 nM) was used to delineate low pH compartments. At comparable time points, IBP-SI exhibited more detectable internalization than SI. Green, low pH compartments (late endosomes and lysosomes); red, ELP nanoparticles; white arrowheads, internalized ELP nanoparticles. Scale bar = 10 µm.

**Figure A3:**
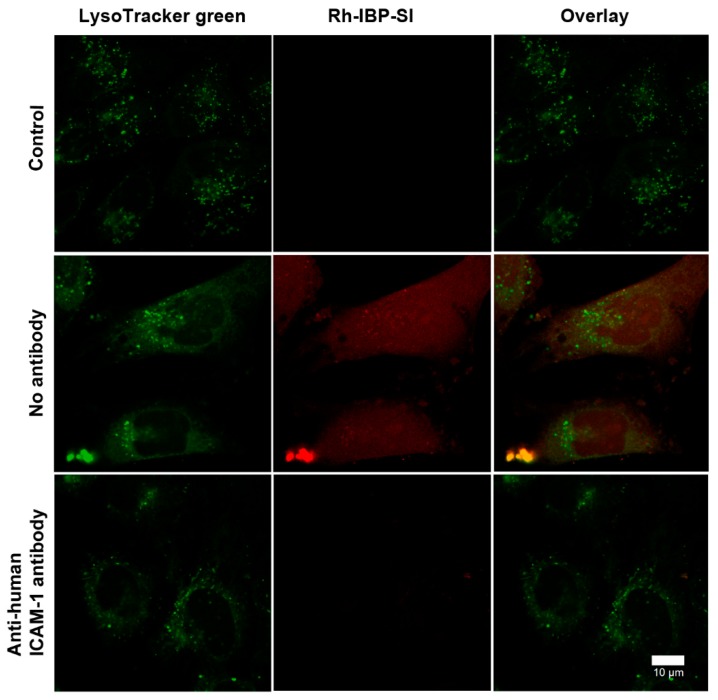
Binding and internalization of IBPSI into HeLa cells is prevented by ICAM-1 antibody. Competition binding assays were performed when Hela cells reached 70% confluence in 35 mm glass-bottomed dishes. Anti-human ICAM-1 antibody was diluted with warm medium at a 10-fold dilution and incubated with Hela cells at 37 °C for 30 min. HeLa cells were then rinsed with warm medium and incubated with rhodamine-conjugated IBP-SI (red, 30 µM) and LysoTracker green (green, 75 nM). After 30 min, the cells were rinsed with fresh warm medium and imaged using confocal microscopy. HeLa cells showed significant reduction in rhodamine signal after cells were incubated with anti-human ICAM-1 antibody. LysoTracker green was used to stain low pH lysosomes inside the cells. Scale bar: 10 µm.

Cross-species reactivity of IBP-SI against human ICAM-1 was also seen. In Appendix A Figure A2, non-transfected HeLa cells incubated with rh-IBP-SI showed accumulation of red puncta as the incubation period increased, relative to those incubated with rh-SI. HeLa cells pretreated with anti-human ICAM-1 antibody exhibited decreased surface binding and uptake of IBP-SI compared to untreated cells (Appendix A Figure A3). These data collectively suggest that IBP-SI is recognized and internalized both by mouse and human ICAM-1 (Figure 3, Figure A2 and Figure A3).

**Figure 4:**
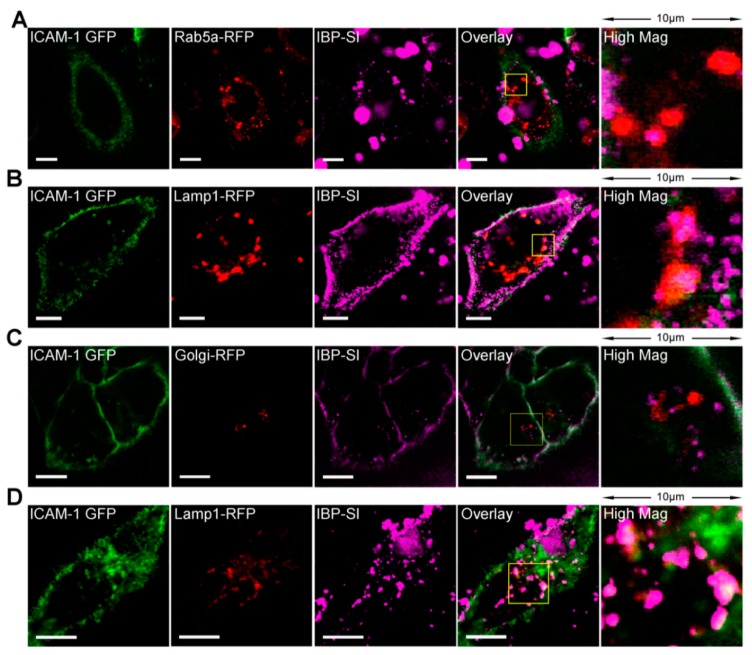
IBP-SI nanoparticles traffic to early endosomes and lysosomes. mICAM-1 expressing HeLa cells transduced with CellLight^®^ (**A**) Rab5-RFP, (**B**) Lamp1-RFP, and (**C**) Golgi-RFP Bacmam 2.0 reagents were pulsed with 30 µM Cy5-labeled IBP-SI for 10 min, with a 45-min chase period prior to imaging. IBP-SI showed apparent regions of co-localization with Rab5-RFP (Panel A, Overlay) and extensive apparent colocalization with Lamp1-RFP (Panel B, Overlay) but was not colocalized with Golgi-RFP (Panel C, Overlay), suggestive of its routing through early endosomes to lysosomes. (**D**) IBP-SI exhibits high co-localization with Lamp1-RFP (Panel D, Overlay) after 3 h of incubation. This experiment was repeated at least twice. Scale bar = 10 µm.

Having demonstrated the cross-species ability of IBP-SI to be internalized more efficiently than SI in cells expressing mouse or human ICAM-1, its intracellular trafficking was assessed utilizing a pulse-chase approach. mICAM-1 transfected HeLa cells were co-transduced with baculoviruses encoding either Rab5a-, Lamp1-, or Golgi-red fluorescent protein (RFP) (red) to mark early endosomes, lysosomes, and Golgi, respectively. IBP-SI was labeled with Cy5 and incubated with cells for 15 min at a concentration of 30 µM before live cell confocal fluorescence microscopy imaging for another 45 min. This analysis revealed that some IBP-SI was co-localized with Rab5-enriched early endosomes after 15 min incubation (Figure 4A). The fluorescent signal of IBP-SI was increasingly observed in lysosomes throughout the entire chase period (Figure 4B) but not in the Golgi (Figure 4C). In addition, analysis by confocal microscopy imaging used to analyze ICAM-1 expressing HeLa cells 3 h after the 1 h chase with Cy5-IBP-SI showed that Cy5-IBP-SI resided almost exclusively in lysosomes (Figure 4D). These findings collectively suggest that IBP -SI bound to ICAM-1 is first sorted into early endosomes and then a fraction of this endocytosed material accumulates in lysosomes. These findings further suggest that IBP-SI binding triggers internalization of ICAM-1, which has the potential to reduce tissue signals for lymphocyte homing.

### 2.3. ICAM-1 Is Significantly Overexpressed in the LG of NOD Mice Compared with Healthy Mice

**Figure 5:**
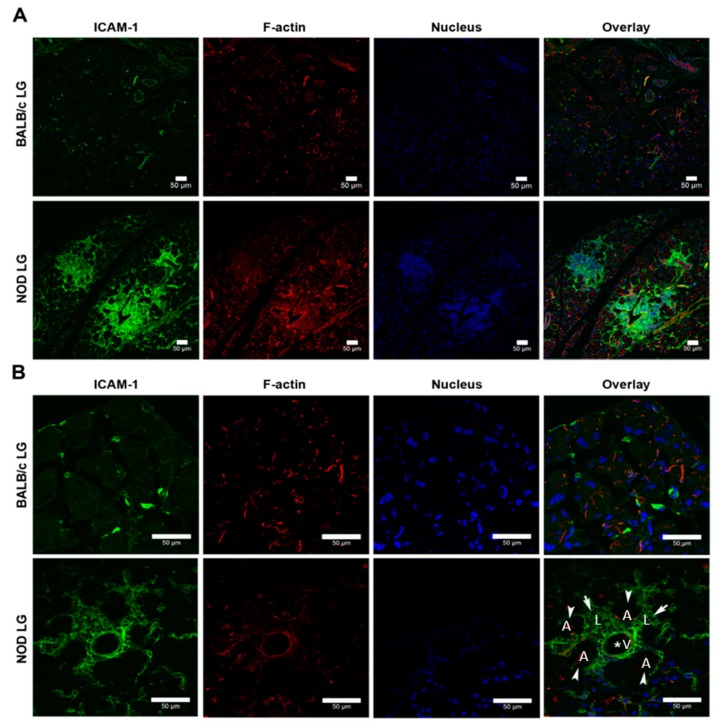
Distribution of mICAM-1 in lacrimal glands (LGs) of BALB/c and NOD mice. Cryosections of LGs from 12-week-old male BALB/c and NOD mice were processed and imaged at low and high magnification using confocal microscopy. mICAM-1 was labeled with goat anti-mouse ICAM-1 primary antibody and AF-488 conjugated donkey anti-goat secondary antibody (green). F-actin was labeled with rhodamine phalloidin (red) and nuclei were labeled with 4′,6-diamidino-2-phenylindole (DAPI) (blue). (**A**) mICAM-1 expression is detected in lymphocytic infiltrates of the LG and correlated with the severity of the inflammation. (**B**) mICAM-1 expression was also observed in vascular endothelium, lymphocytes, and basolateral membranes of LG epithelial cells next to lymphocytes. White arrowheads (A): LG acini; white arrows (L): lymphocytes; * (V): vascular lumen.

Male NOD mice have been reported to fully develop the autoimmune dacryoadenitis characteristic of SS, manifested as a significant lymphocytic infiltration in the LG by 12 weeks of age [33]. A previous analysis of ICAM-1 gene and protein expression showed that ICAM-1 is increased by 3.5-fold in NOD mouse LG [20]. ICAM is also overexpressed in the LG of lymphoproliferative, MRL-*lpr* mice, another mouse model of SS [19]. LG are comprised of 85% by mass LG acinar cells (LGACs; polarized epithelial cells responsible for transport, production and secretion of tear proteins), as well as vasculature, ductal epithelium, and lymphocytes. Immunofluorescence was used to determine the distribution of ICAM-1 protein in these cell types in BALB/c and NOD LG. Lymphoid foci are detected in NOD LGs but not BALB/c LGs, and ICAM-1 was enriched in these foci (Figure 5A). Higher magnification images revealed that ICAM-1 was overexpressed in basolateral membranes of LGACs from NOD mice, vasculature, and individual lymphocytes. ICAM-1 could be also be detected on sparsely distributed cells in BALB/c LGs (Figure 5B).

**Figure A4:**
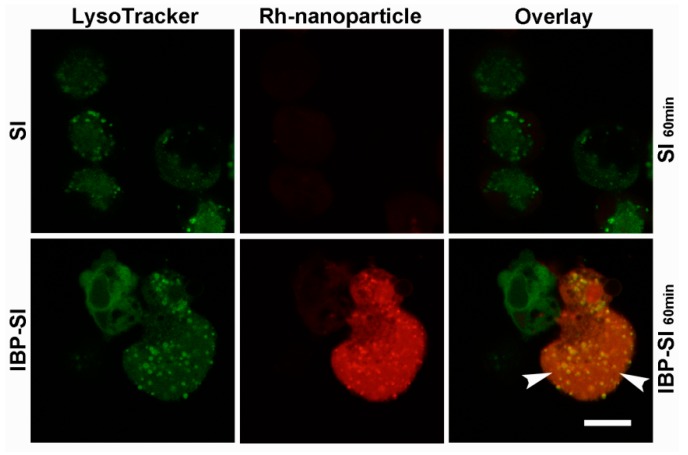
Addition of mouse ICAM-1 targeting peptide to SI enhances the ICAM-1 targeting specificity and internalization of SI in NOD LG acini. LGs from 12–14 week male NOD mice were removed and minced into 1 mm^3^ pieces with scalpels and sequentially grown in serum-free culture media containing 5 µg/mL laminin, 0.1 µM carbachol, and 1 nM thyroxine. Cells were seeded on a Matrigel^TM^ coat for live cell imaging on 35 mm glass-bottomed dishes at 6 × 10^6^ cells per dish and incubated at 37 °C for 2–3 h prior to experiments. Acini were then treated with 30 μM rhodamine-labeled (rh)-SI or IBP-SI at 37 °C for 1 h. Rh-IBP-SI (red) exhibits significantly higher surface binding, internalization, and co-localization with LysoTracker (green), a biomarker for low pH compartments, in NOD LG acini than its SI counterpart. White arrowheads indicate the co-localization of IBP-SI and LysoTracker. Scale bar = 10 µm.

Consistent with the increased expression of ICAM-1 in the LGACs of NOD mice, internalization of IBP-SI, but not SI, was also observed when ex vivo cultured primary LGACs from NOD mice were incubated with rh-IBP-SI and rh-SI (Appendix A Figure A4).

### 2.4. Inhibition of Lymphocyte Binding to ICAM-1 Enriched bEnd.3 Cells by IBP-SI

**Figure 6:**
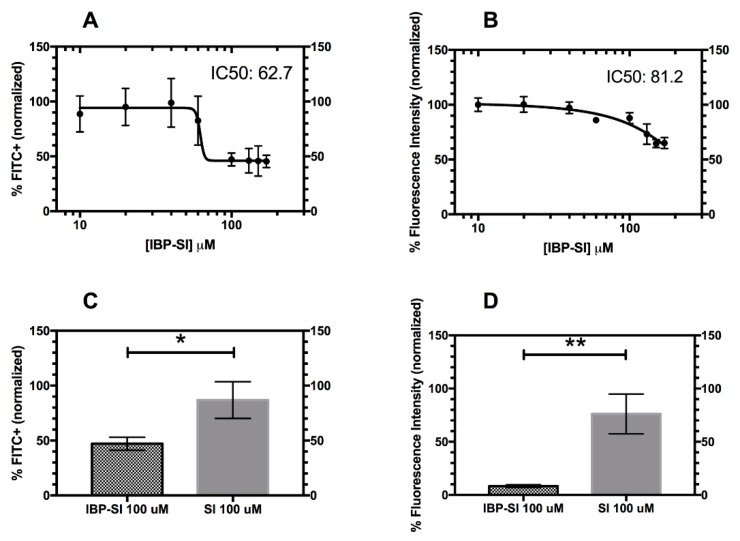
IBP-SI significantly inhibits the binding of splenocytes to ICAM-1 overexpressing endothelial cells. IBP-SI shows concentration-dependent inhibition of Phorbol 12-myristate 13-acetate (PMA)-induced splenocyte binding to ICAM-1-overexpressing bEnd.3 cells at 37 °C, as assessed by detection of Carboxyfluorescein succinimidyl ester (CFSE)-labeled splenocyte fluorescence by flow cytometry (**A**,**C**) and a fluorescence plate reader (**B**,**D**). (**A**) The IC_50_ of IBP-SI calculated using flow cytometry. The amount of splenocytes adhering to bEnd.3 cells was measured by flow cytometry and normalized to splenocyte binding to cells in the absence of added ELP, which was defined as 100%. (**B**) The IC_50_ of IBP-SI was identified by a plate reader assay. (**C**) PMA-induced splenocytes, labelled with CFSE, were incubated with a Tumor Necrosis Factor (TNF)-α stimulated bEnd.3 monolayer, with a confluency above 80%, in the presence of 100 µM IBP-SI or SI for 45 min at 37 °C. (**D**) PMA-induced splenocytes were incubated with a TNF-α stimulated bEnd.3 monolayer, at a confluency above 80%, in the presence of 100 µM IBP-SI or SI for 45 min at 37 °C. Adhesion was confirmed by retention of fluorescence in a plate-based assay. The percentage of fluorescence intensity relative to no-ELP treatment control, defined as 100%, is plotted under each condition (Equation (1)). Each treatment was performed in triplicate wells three times and plotted as the overall mean ± SD for (**A**–**D**). An IBP-SI and SI concentration above the IC_50_ was used to observe maximal inhibition for (**C**) and (**D**). * *p* < 0.05, ** *p* < 0.01.

The lymphocyte function-associated antigen-1/intercellular adhesion molecule-1 (LFA-1/ICAM-1) interaction plays an important role in lymphocytic adhesion and trans-vessel migration. The induction of ICAM-1 in LG in NOD mice with autoimmune dacryoadenitis seen in Figure 5 is likely involved in homing of immune cells into the LG associated with autoimmune inflammation. To assess whether IBP-SI disrupts the interaction between these two receptors, bEnd.3 cells were induced with TNF-α to express high levels of mICAM-1 as a source of ICAM-1, and mouse splenocytes were stained with Carboxyfluorescein succinimidyl ester (CFSE) as a source of LFA-1 enriched cells. Their interactions were probed using an in vitro assay. Briefly, splenocytes were stimulated with Phorbol 12-myristate 13-acetate (PMA) to activate LFA-1 before incubation with bEnd.3 cells [34,35]. IBP-SI at various concentrations was incubated with splenocytes and then added to wells containing confluent bEnd.3 cells induced to express mICAM-1. SI was used as a control for non-specific effects of the ELP nanoparticle, as it was not expected to bind to mICAM-1. A concentration-dependent inhibition of splenocyte adherence was observed in the flow cytometry assay for IBP-SI, with an IC_50_ of 62.7 µM IBP-SI (Figure 6A), while the plate reader assay showed that IBP-SI also inhibited splenocyte adherence with an IC_50_ of 81.2 µM (Figure 6B). Using 100 µM concentrations of IBP-SI and SI, which slightly exceed the IC_50_, IBP-SI significantly inhibited splenocyte binding to an average of 47.1% (*p* = 0.017). As a negative control, SI showed little reduction of splenocyte binding at 86.9% (Figure 6C) as determined by flow cytometry. Similarly, we observed that IBP-SI significantly inhibited the adhesion of fluorescently labeled splenocytes to an average of 8.3%, while SI showed minimal reduction at 76.2% (*p* = 0.003) (Figure 6D). This further demonstrates the ability of IBP-SI to specifically target ICAM-1 expressing cells while dually disrupting splenocyte adherence.

### 2.5. IBP-SI Inhibits the Activation and Proliferation of T-Cells

**Figure 7:**
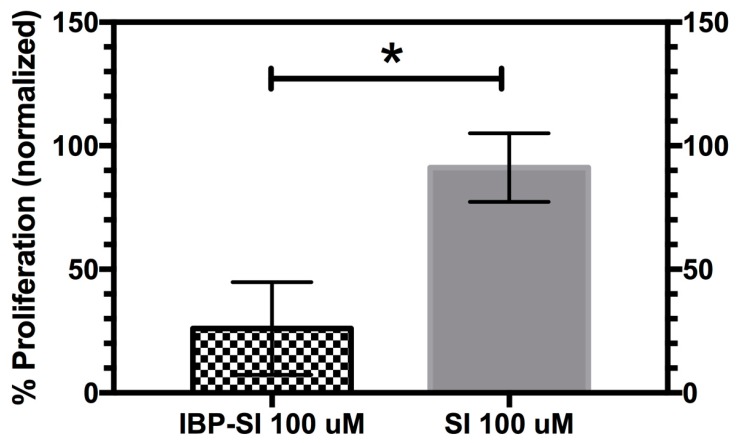
IBP-SI significantly reduces the proliferation of CD4+ T-cells relative to SI. CH27 cells treated with 15 µM moth cytochrome c (MCC) antigenic peptide were incubated with CFSE-labelled CD4+ T-cells in the presence of 100 µM IBP-SI or SI. After 5 days of incubation, FITC+ cells were analyzed by flow cytometry and the results were fitted into a proliferation model with FlowJo (v10.6.2). The percentage of proliferation compared to generation 0 calculated from the fitting model for the IBP-SI and SI-treated groups was plotted after normalization to that from non-ELP treated controls. Each treatment was done in triplicate wells, and the same experiments were repeated three times. Data is plotted as mean ± SD. * *p* < 0.05

The ICAM-1/LFA-1 interaction not only plays an important role in leukocyte adhesion and migration, but also acts as an important co-stimulatory factor for T-cell activation by antigen presenting cells [36,37]. Interfering with the LFA-1/ICAM-1 interaction can impair T-cell activation, proliferation and differentiation [38], which is not mitigated by boosting the interaction between T-Cell Receptor (TCR) and Major Histocompatibility Complex MHC complexes [39]. Therapies targeting the ICAM-1/LFA-1 interaction have been shown to have positive effects in several eye diseases such as dry eye disease and allergic conjunctivitis [40,41]. Here, we tested whether IBP-SI can inhibit the activation and proliferation of T-cells through its ability to disrupt the ICAM-1/LFA-1 interaction. CH27 cells are a mouse B-cell line widely used as an antigen presenting cell line. CH27 cells can stimulate the activation and proliferation of T-cells in the presence of the moth cytochrome C (MCC) antigenic peptide [42,43]. We found that proliferation of T-cells isolated from the mouse spleen and stimulated with MCC-loaded CH27 cells was inhibited by IBP-SI to 26.1 ± 18.7% (*p* = 0.01) of that seen in the untreated mixture, whereas no significant inhibition (91.2 ± 13.9%) was observed with SI (Figure 7).

## 3. Discussion

This manuscript evaluates a multivalent ICAM-1 antagonist nanoparticle, IBP-SI, which is internalized in ICAM-1 expressing cells, inhibits the adhesion of leukocytes to endothelial cells, and inhibits T-cell activation and proliferation in vitro. This multivalent nanoparticle represents a new tool for probing ICAM-1/LFA-1 interactions in vitro. The mICAM-1 specific peptide used in this study was identified by screening a phage displayed peptide library, which is also multivalent, against mICAM-1 transfected COS-7 cells. It retained the highest avidity for domain-1 of mICAM-1 and was reported to block LFA-1 interactions during antigen presentation [25]. Several ICAM-1 affinity peptides, mostly generated by phage display techniques, and ICAM-1 targeting nanocarriers have been developed in the past to block leukocyte–endothelial adhesion or intercellular adhesion of immune cells during antigen presentation in vitro and in vivo [44,45]. Some of these have shown an interspecies targeting ability for two or more species. Our work demonstrates that the IBP peptide sequence has cross-species reactivity in mouse and human ICAM-1.

Multiple groups have taken diverse approaches to targeting therapeutic particles using ICAM-1 as a molecular marker and/or to simply target ICAM-1 to inhibit its molecular interactions in driving inflammatory responses. The Muro and Muzykantov groups have used targeted therapeutics to endothelial ICAM-1 in vitro and in vivo for treatment of lysosomal storage diseases, acute and chronic respiratory diseases, and other vascular diseases [32,46,47,48]. Zhang et al. have developed a cyclic LABL(cLABL)-conjugated Poly Lactic-*co*-Glycolic Acid (PLGA) nanoparticle approximately 244 nm in diameter which binds both human epithelial and endothelial ICAM-1 [45,49]. cLABL is a cyclo(1,12)PenITDGEATDSGC peptide derived from the α_L_-integrin I domain of LFA-1 which blocks T-cell adhesion and reduces lymphocytic infiltration in islet endothelium [50]. By electrostatic adsorption of targeting antibodies onto the surface, polystyrene latex microspheres with particle sizes ranging from 100 nm to 300 nm in diameter were selectively delivered to mouse or human ICAM-1 or to other adhesion molecules. Of note, a 17-mer linear peptide derived from the ICAM-1-binding sequence of fibrinogen [44] that shares high sequence homology across human, mouse, and chimpanzees can, when adsorbed to polystyrene nanoparticles, selectively target both human and mouse ICAM-1 similarly to IBP-SI.

Multiple antagonizing peptides have been developed to disrupt the interaction between ICAM-1 and LFA-1. Short peptides derived from the LFA-1 binding domain on ICAM-1 or the ICAM-1 binding domain on LFA-1 showed 23–52% inhibition of LFA-1/ICAM-1-dependent intercellular adhesion at 250 μM. Those peptides also inhibited lymphocyte proliferation in a mixed cell reaction by around 60% at 600 μM [51,52]. Another ICAM-1 antagonizing peptide discovered by phage display (LLRMRSIC) showed inhibition of ICAM-1 mediates the adhesion of neutrophils to endothelial cells at a concentration of 2 mM [53]. In comparison, IBP-SI inhibits ICAM-1 mediated cellular adhesion with IC_50_ around 70 μM and inhibits the proliferation of T-cells by around 70% at 100 μM. This is a significant improvement compared to other reported peptides.

Xiidra^®^ (5% Lifitegrast ophthalmic solution), which inhibits the LFA-1/ICAM-1 interaction by targeting the I-domain of LFA-1, was approved in 2016 as a treatment for dry eye disease. In phase II and III clinical trials, dry eye patients treated with Lifitegrast showed significant improvement in clinical symptoms and ocular surface health when compared with patients that received the placebo [54,55]. This suggests that other strategies for the inhibition of ICAM-1/LFA-1 interactions, including the eventual in vivo use of IBP-SI, could also be effective strategies for the treatment of dry eye disease. It is notable that this same SI nanoparticle backbone has already been used as a rapamycin drug carrier with therapeutic efficacy in a murine model of SS [7].

IBP-SI has several unique features compared to other ICAM-1 targeted nanoparticles [45,47,56]. The protein sequence and size of ELPs can be tuned precisely through genetic engineering of recombinant plasmid in a way that is not achievable by synthetic polymers. ELPs are also characterized by high biocompatibility and biodegradability and low immunogenicity, permitting their safe breakdown into peptides and amino acids that are easily cleared from the body [57]. The major limitations of antibody-targeted therapeutics has been their immunogenicity induced from the Fragment crystallizable regions (F_c_ regions) of IgGs [58] as well as the non-specific uptake of antibodies by the reticuloendothelial system [59]. It is widely believed that short peptides with sequences less than 20 amino acids in length should be less immunogenic relative to native proteins [60]. Hence, conjugating a 16-mer ICAM-1 targeted peptide to SI may be expected to arouse minimal host immune responses after long-term in vivo treatment with this ELP nanoparticle.

In summary, IBP-SI constitutes a unique new tool which may be utilized in vitro to probe the role of ICAM-1/LFA-1 interactions in physiological or pathophysiological processes. As a genetically encoded protein polymer constructed on the ELP backbone, its ease of production, biocompatibility and lack of immunogenicity may offer advantages relative to alternative ICAM-1 targeting strategies moving into in vivo studies. The investigation of its therapeutic potential utilizing local and systemic administration for autoimmune dacryoadenitis in SS will be an important next step.

## 4. Materials and Methods

### 4.1. Materials and Reagents

NHS-rhodamine was from Thermo Fisher Scientific (Rockford, IL, USA). Sulfo-Cy5 NHS ester was from Lumiprobe Corp. (Hallandale Beach, FL, USA). Alexa Fluor^®^ 488 donkey anti-goat IgG, DAPI (4′,6-diamidino-2-phenylindole dihydrochloride), rhodamine-phalloidin, CellLight^®^ RFP-Rab5a, RFP-Lamp1, and RFP-Golgi BacMam2.0 reagents were from Life Technologies (Grand Island, NY). HeLa and bEnd.3 cells were from American Type Culture Collection (ATCC) (Manassas, VA, USA). The CH27 cell line was a kind gift from Jianming Xie (University of Southern California Manassas). The plasmid expressing mouse ICAM-1 GFP was from Origene (Rockville, MD, USA). Mouse ICAM-1 (mICAM-1) polyclonal antibody was from R&D Systems (Minneapolis, MN, USA). Glass-bottomed dishes (35 mm) were from MatTek Corp. (Ashland, MA, USA). The 5-and 6-carboxyfluorescein diacetate succinimidyl ester kit (CFSE), Alexa Fluor^®^ 700 anti-mouse CD3 antibody and MojoSort™ CD4+ T-cell isolation kits were from Biolegend (San Diego, CA, USA). Recombinant mouse TNF-α was from Abcam (Cambridge, UK). Moth cytochrome C (83-103) was from GenScript (Piscataway, NJ, USA). Other reagents were from standard suppliers.

### 4.2. Animals

BALB/c, C57BL/6J and NOD (SS disease model) breeding pairs were purchased from Charles River Laboratories (Wilmington, MA, USA), The Jackson Laboratory (Bar Harbor, ME, USA) and Taconic Farms (Hudson, NY, USA), respectively. Animals were bred in the University of Southern California (USC) vivarium. All animal procedures were approved by the USC Institutional Animal Care and Use Committee under protocols 11788 (first approved for these studies Jan 2012) and 10547 (first approved for these studies Nov 2008), both protocols have been revised and renewed yearly after that. All procedures were performed in accordance to the Guide for Care and Use of Laboratory Animals, 8th edition [61]. For analysis of LG in BALB/c and NOD mice, all mice used in this study were males, aged 12–14 weeks. For immunofluorescence labeling of ICAM-1, LGs were removed after mice were euthanized via intraperitoneal injection with 55 mg ketamine and 14 mg zylazine per kilogram of body weight, followed by cervical dislocation. For isolation of splenocytes, the spleens from female C57BL/6J mice were removed after the mice were euthanized as described above.

### 4.3. Construction of IBP-SI ELP Nanoparticles

The plasmid pET25b(+) encoding the ELP diblock copolymer, SI, was synthesized using plasmid recursive directional ligation as described previously [62]. Sense and antisense murine ICAM-1 specific peptide sequences, identified by phage display selection [25], were synthesized with NdeI (5′) and BamHI (3′) overhangs (Integrated DNA Technologies Inc., Coralville, IA): sense (5′-TATGGGTTTCGAAGGCTTCTCGTTCCTCGCATTCGAAGACTTCGTATCATCAATAGGTTACTGATCTCCTCGG-3′) and antisense (5′-GATCCCGAGGAGATCAGTAACCTATTGATGATACGAAGTCTTCGAATGCGAGGAACGAGAAGCCTTCGAAACCCA-3′). Complementary oligonucleotides were heated at 95 °C for 2 min and cooled to room temperature for 3 h. The annealed oligonucleotides were ligated into the pET25b(+) vector, previously digested with NdeI and BamHI. The SI gene was then ligated downstream of the murine ICAM-1 specific sequence using BseRI and BssHII cutting sites in both plasmids. The correct ligation clones were confirmed by DNA diagnostic digestion and DNA sequencing. ELPs were expressed in the BLR (DE3) *E. coli* strain and purified by induction of the ELP-mediated phase separation. The purity of ELP fusion proteins was determined by running 50 µg of samples on a 4–20% SDS-PAGE gel stained with 10% (*w*/*v*) copper chloride. Protein concentrations were determined by measuring the absorbance of protein polymers at 280 nm and calculated using the Beer-Lambert law (*ε*_SI/IBP-SI_ = 1285 M^−1^ cm^−1^). Protein molecular mass was further confirmed by MALDI-TOF mass spectrometry (AXIMA Assurance, Shimadzu, Kyoto, Japan).

### 4.4. Characterization of IBP-SI Phase Transition Behavior and Nanoparticle Formation

The temperature–concentration phase diagram of IBP-SI ELP was determined by optical density measurements at 350 nm using a DU800 UV-vis spectrophotometer (Beckman Coulter, Brea, CA, USA). *T_t_* was defined as the temperature point showing 50% maximal turbidity. Self-assembly and hydrodynamic radius (R_h_) of ELP nanoparticles was measured using DLS in a DynaPro-LSR Plate Reader (Wyatt Technology, Santa Barbara, CA, USA). For DLS measurements, samples were prepared at 25 μM in PBS and filtered through a filter with a 0.02 μm pore size at 4 °C. Each sample (90 μL) was applied to a pre-chilled 384 well microplate, centrifuged at 4 °C to remove air bubbles, and covered with 20 μL of mineral oil to prevent evaporation. DLS data were recorded at regular temperature intervals (1 °C) as solutions were heated from 10 °C to 37 °C. The results were fitted to a cumulant algorithm based on the sum-of-squares value and analyzed with a Rayleigh sphere model. *T_t_*_1_, the critical micelle temperature, was defined as the lowest temperature at which the R_h_ is significantly greater than the averaged monomer R_h_.

### 4.5. Fluorescent Labelling of ELP Nanoparticles

For fluorescent visualization, SI and IBP-SI ELPs were conjugated with NHS-rhodamine ester or Sulfo-Cy5 NHS ester via the chemical covalent crosslinking of the fluorophore to the lysine at the N-terminus of ELP polypeptides. Conjugation reactions were performed in 100 mM borate buffer (pH 8.5) at 4 °C overnight. Excess fluorophore was removed by size exclusion chromatography on a pre-packed G-25 desalting column (GE Healthcare, Piscataway, NJ, USA).

### 4.6. In Vitro Cell Uptake

HeLa cells were grown in a 35 mm glass-bottomed plate until at 90% confluence or greater. Transfection of mICAM-1 GFP by Lipofectamine 2000^®^ was performed according to the manufacturer’s recommendation. Briefly, cells were treated with a mixture of mICAM-1 GFP plasmid (μg) and Lipofectamine 2000^®^ (μL) at a ratio of 1:2 at 37 °C for 6 h followed by a wash with PBS, and maintained in complete medium at 37 °C for another 36–48 h prior to uptake and intracellular trafficking experiments. For uptake studies, mICAM-1 expressing HeLa cells were incubated with 30 µM of rhodamine-labelled (rh)-SI or -IBP-SI at 37 °C for 30 min and 120 min. Cells were then rinsed with warm PBS three times, maintained in fresh culture medium, and imaged by confocal fluorescence microscopy. To follow the intracellular trafficking of internalized IBP-SI, mICAM-1-expressing HeLa cells were transduced with recombinant baculovirus expressing RFP-Rab5a, RFP-Lamp-1, or RFP-Golgi. After 16–18 h of transduction, cells were incubated with 30 µM of Cy5-IBP-SI at 37 °C for 10 min, rinsed with warm PBS three times, and imaged for another 45 min. Images were acquired using a Zeiss laser scanning microscope 510 Meta NLO or an LSM 800 confocal imaging system (Carl Zeiss, Thornwood, NY, USA).

### 4.7. Immunofluorescence of Mouse LG

Immunofluorescence labeling of mouse LG was performed as previously described [20]. Briefly, LGs from NOD and BALB/c mice were fixed, embedded and cut into 5 µm sections. For immunofluorescence labeling, LG sections were quenched with 50 mM NH_4_Cl, permeabilized with 0.1% Tx-100, and blocked with 1% bovine serum albumin (BSA). The resulting slides were then incubated with goat-anti mICAM-1 primary antibody at 4 °C overnight followed by incubation with donkey-derived Alexa Fluor 488-conjugated anti-goat IgG secondary antibody at 37 °C for 1 h before analysis by confocal microscopy.

### 4.8. Splenocyte Isolation, Labelling and Quantitation

Freshly isolated spleens from C57BL/6 female mice were homogenized by pressing the tissue through a 70 µm filter into a 50 mL conical tube while washing with red blood cell lysing buffer (0.15 M NH_4_Cl, 1 mM NaHCO_3_, 0.1 mM Ethylenediaminetetraacetic acid EDTA). The remaining cell suspension was spun down at 437× *g* for 10 min, resuspended in PBS and counted using a TC20 automated cell counter (Bio-Rad Laboratories, Inc. Hercules, CA). Splenocytes were labelled with 2 µM CFSE and incubated at 37 °C for 20 min, away from light. The remaining free label was then quenched with 5 mL of bEnd.3 culture media, in accordance with the manufacturer’s guidelines. Splenocytes were treated with PMA at 10 ng/mL for 30 min to induce activation of LFA-1 on cell surfaces prior to incubation with bEnd.3 cells [20].

### 4.9. In Vitro Static Adhesion Assay

bEnd.3 cells were cultured in Dulbecco’s Modified Eagle’s Medium (DMEM), 10% fetal bovine serum, 1× penicillin/streptomycin and 2 mM L-glutamine at 37 °C in a 5% CO_2_ incubator. bEnd.3 cells were incubated with mouse TNF-α at 10 ng/mL overnight the day before the assay to induce ICAM-1 expression. IBI-SI, at concentrations from 10 to 150 µM, or SI at 100 µM were diluted into adhesion solution (PBS containing 0.5% BSA, 2 mM MgCl_2_ and 1 mM CaCl_2_) and incubated with 1 × 10^6^ CFSE-labelled splenocytes, and then added to TNF-α induced bEnd.3 cells grown on clear 24-well flat bottom tissue culture plates at 37 °C 5% CO_2_ for 45 min. Non-adherent cells were aspirated gently and each well was washed twice with 250 µL of wash solution (PBS with 2 mM MgCl_2_ and 1 mM CaCl_2_). Adherent cells were then trypsinized, pelleted by centrifugation, and resuspended in Fluorescence-activated cell sorting FACS buffer (PBS with 0.5% BSA and 1 mM EDTA) prior to analysis on an LSR2 flow cytometer (BD Biosciences). Data were analyzed using FlowJo software and the percentage of fluorescent splenocytes associated with ICAM-1-overexpressing bEnd.3 cells under each treatment condition was expressed as a percentage of non-ELP-treated splenocytes binding to cells, defined as 100%. Similarly, IBP-SI and SI effects on ICAM-1 overexpressing bEnd.3 cells and PMA-induced CFSE-labelled splenocyte binding were assessed using a plate assay. Briefly, bEnd.3 cells seeded in a 96-well flat black bottom plate were stimulated with TNF-α as above. IBP-SI at varying concentrations (10–150 µM) or SI at 100 µM in adhesion solution were incubated with 1 × 10^5^ CFSE-stained, PMA-induced splenocytes at 37 °C in 5% CO_2_ for 45 min per well, as previously described. Non-adherent cells were gently aspirated, and wells were washed twice with 50 µL of wash solution. Then, 100 µL of wash solution was added to each well. Fluorescence was measured with excitation at 485 nm and emission at 535 nm using a plate reader (SpectraMax iD3; Molecular Devices) and SoftMax Pro (Molecular Devices). Each condition utilized triplicate wells. The fluorescence associated with splenocyte retention in wells containing ICAM-1-overexpressing bEnd.3 cells under each treatment condition was expressed as a percentage of fluorescence associated with non-ELP-treated splenocytes binding to cells, defined as 100%, following Equation (1):

% normalized = (treated−background)/(non-ELP treated−background)*100%(1)

### 4.10. Antigen Presentation Assay

For the isolation of CD4+ T cells, splenocytes from C57BL/6J female mice were isolated as described above. A biotin antibody cocktail (anti-CD8a, CD11b, CD11c, CD19, CD24, CD45R/B220, CD49b, CD105, 1-A/I-E (MHC-II), TER-119/Erythroid) (BioLegend) was incubated with the cell suspension at 10 µL per 1 × 10^7^ cells in 100 µL of buffer on ice for 15 min. Following the incubation, streptavidin-coated magnetic beads (BioLegend) were added in an equal volume and incubated for 15 min again on ice. The solution was left to rest for 5 min in a magnetic stand (Stemcell) at room temperature. The supernatant containing unlabeled CD4+ T-cells was dispensed and counted on an automated cell counter. CD4+ T-cells were then labelled with CFSE, as previously described. CH27 B cells were grown in Roswell Park Memorial Institute Medium RPMI 1640 supplemented with 2 mM L-Glutamine, 1× penicillin/streptomycin and 10% FBS. Prior to the mixed cell reaction, they were counted using a TC20 automated cell counter and spun down at 70× *g* for 10 min. They were then resuspended in CH27 media containing MCC peptide (15 µM) at 1 × 10^5^ cells per 50 µL and incubated for 30 min at 37 °C in 5% CO_2_. To identify the dose at which inhibition of T-cell proliferation was optimized, dilutions of IBP-SI (200 µM) and SI (200 µM) in CH27 medium containing CFSE-labelled CD4+ T-cells were prepared in clear 96-well round bottom tissue culture plates. CH27 cell suspension containing MCC (50 µL) and 50 µL of ELP plus CD4+ T-cells were co-incubated at 37 °C in 5% CO_2_ for 5 days. Non-ELP treated CD4+ T-cells were also incubated 1:1 with MCC primed CH27 B-cells as a positive control. A final concentration of 1 × 10^5^ CH27 B-cells and 1 × 10^5^ CD4+ T-cells was used per treatment, and ELP concentrations of 100 µM IBP-SI and SI were chosen for further investigation (a dose slightly above that of the IC_50_). Each treatment was done in triplicate wells. After 5 days, each well was collected into separate 1.7 mL tubes and counted with a cell counter. Alexa Fluor 700 anti-mouse CD3 antibody at 0.5 µg per million cells was diluted in PBS and added to each tube. After 30 min incubation on ice, cells were pelleted down at 20 °C at 2655× *g* for 8 min and resuspended in FACS buffer. Samples were read using FITC and APC-Cy7 channels for CFSE-labelled and CD3-labelled T-cells, respectively, on an LSR2 flow cytometer. Data were analyzed using FlowJo software. Data are shown as the percentage of cell proliferation for each treatment normalized to total proliferation from day 0 to day 5 for non-ELP treated positive controls, defined as 100%.

### 4.11. Statistics

All data were expressed as mean ± SD. Means from both groups were analysed using an unpaired two-tailed Student’s t-test. A *p* value of less than 0.05 was considered statistically significant.

## 5. Conclusions

This manuscript describes a multivalent protein polymer that targets ICAM-1 in vitro and is internalized through ICAM-1 mediated endocytosis. As demonstrated using different measures of ICAM-1 and LFA-1 engagement, IBP-SI, but not the parent SI nanoparticle, inhibited the interaction between these key effectors of inflammation. Furthermore, IBP-SI, but not SI, prevented co-activation during antigen presentation, which is associated with T-cell proliferation. Future studies will explore the further utility and enhancement of this probe in vivo as well as its potential for therapeutic effect.

## Figures and Tables

**Table 1:** Biophysical characteristics of evaluated ELP protein–polymers.

Label	Amino Acid Sequence ^a^	*T_t_*_1_^b^ (°C)	*T_t_*_2_^c^ (°C)	Expected MW ^d^ (kDa)	Measured MW ^e^ (kDa)	Hydrodynamic Radius ^f^ (nm)
SI	MG(VPGSG)_48_(VPGIG)_48_Y	25.5	73.8	39.6	39.5	23.6 ± 0.4
IBP-SI	MG**FEGFSFLAFEDFVSSI**G (VPGSG)_48_(VPGIG)_48_Y	25.7	46.9	41.5	41.4	21.9 ± 0.6

^a^**Underlined bold**: mouse ICAM-1 targeting peptide identified by phage display screening [25]. ^b^ Critical micelle temperature (25 μM, pH 7.4) determined by optical density measurements at 350 nm. ^c^ Bulk phase temperature (25 μM, pH 7.4) determined by optical density measurements at 350 nm. ^d^ Expected MW was calculated using DNASTAR Lasergene Editseq (Madison, WI). ^e^ Observed MW ([M+H]+) was determined by MALDI-TOF-MS. ^f^ Hydrodynamic radius between *T_t_*_1_ and *T_t_*_2_ in phosphate-buffered saline (PBS; 25 µM, pH 7.4) was measured by Dynamic Light Scattering (DLS) and is expressed as mean ± standard deviation (SD) (*n* = 10).

**Figure A1:**
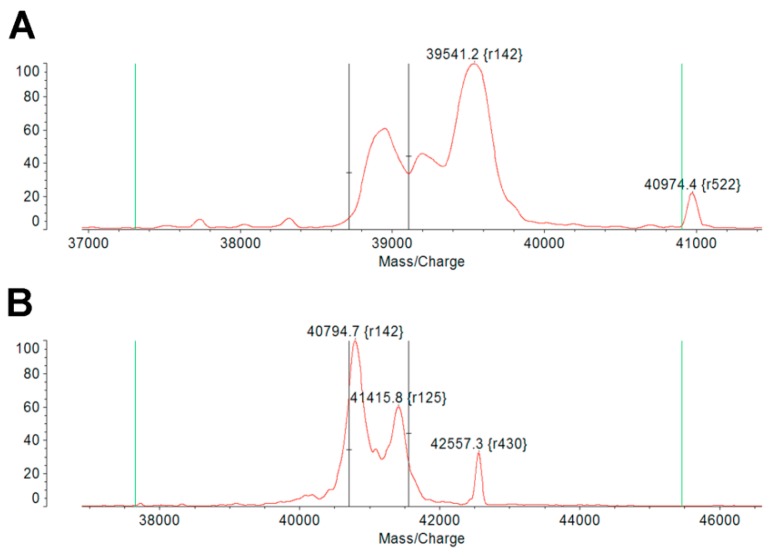
MALDI-TOF mass spectra of (**A**) SI and (**B**) IBP-SI.

## Abbreviations

ATCC CFSE DAPI	American Type Culture Collection carboxyfluorescein diacetate succinimidyl ester 4′6-diamindino-2-phenylindole
DLS EDTA	dynamic light scattering ethylenediaminetetraacetic acid
ELP FACS Fc	elastin-like polypeptide fluorescence-activated cell sorting fragment crystallizable region
ICAM-1	intercellular adhesion molecule-1
IBP-SI IL-1	ICAM-1 binding peptide S48I48 interleukin-1
LFA-1	lymphocyte function-associated antigen 1
LG	lacrimal gland
LGAC	lacrimal gland acinar cell
MCC	moth cytochrome C
MEM MHC	Modified Eagle’s Medium major histocompatibility complex
mICAM-1	mouse ICAM-1
NOD PLGA	non-obese diabetic poly lactic-co-glycolic acid
PMA RFP	phorbol 12-myristate 13-acetate red fluorescent protein
rh RPMI SG	rhodamine roswell park memorial institute medium salivary gland
SI	S48I48
SS TCR	Sjögrens’ syndrome T cell receptor
TNF-α	tumor necrosis factor-α

## Appendix A

**Table A1:** Temperature sensitive phase-transition parameters of SI and IBP-SI.

Constructs	Temperature-Concentration Phase	Diagram
	Slope, m (°CLog(µM))	Intercept, b (°C)
SI (*T_t_*_1_)	−3.7 ± 0.3	32.4 ± 0.4
SI (*T_t_*_2_)	−4.0 ± 0.5	81.1 ± 0.8
IBP-SI (*T_t_*_1_)	−3.9 ± 0.1	31.4 ± 0.2
IBP-SI (*T_t_*_2_)	−4.1 ± 0.3	52.4 ± 0.4
